# Phenotypic Variability and Genetic Diversity of *Phragmites australis* in Quebec and Kashmir Reveal Contrasting Population Structure

**DOI:** 10.3390/plants9101392

**Published:** 2020-10-20

**Authors:** Gowher A. Wani, Manzoor A. Shah, Honoré Tekeu, Zafar A. Reshi, Alain R. Atangana, Damase P. Khasa

**Affiliations:** 1 Department of Botany, University of Kashmir, Srinagar 190006, Jammu & Kashmir, India; mashah@uok.edu.in (M.A.S.); zreshi@kashmiruniversity.ac.in (Z.A.R.); 2 Centre for Forest Research (CEF) and Institute for Integrative and Systems Biology (IBIS), Université Laval, Québec, QC G1V0A6, Canada; honore.tekeu.1@ulaval.ca (H.T.); or A.Atangana@cgiar.org (A.R.A.); Damase.Khasa@ibis.ulaval.ca (D.P.K.); 3 Department of Plant Biology, Faculty of Science, University of Yaoundé, IPO Box 812 Yaoundé, Cameroon; 4 World Agroforestry, West and Central Africa Region, Cocody, Angré 7ème Tranche B.P. 2823, Abidjan 08 BP 2823, Cote D’Ivoire

**Keywords:** common reed, CpDNA microsatellite loci, ecological competitive success, haplotypes, phenotypic and genetic differentiation, plant invasion

## Abstract

The origin of differences in traits influencing competitive success between invasive and native wild populations of alien species is subject of debate. Herbarium-based information sources from 2005 onwards about nativity and distributional range of *Phragmites australis* were used to survey putative native populations of the species in Quebec, and chloroplast DNA (cpDNA) PCR-RFLP analyses identified only one native population, whereas the same analyses revealed that the Kashmir populations are invasive. We compared the native population of *P. australis* in Quebec (QN), ten populations invasive to Quebec (QE), and five populations invasive in Kashmir, India (KE) using morphometric traits. Using nine cpDNA microsatellite loci, we also compared nine KE populations, ten QE populations, and the QN population. Phenotypic variation was observed among and within populations. Only dry mass of flowers varied across regions. Characterization of morphotypes defined three distinct haplotypes. A bimodal distribution of stem diameter (SD), internode length (IL), leaf length (LL), and leaf width (LW) suggests that a major gene may control growth traits or occurrence of co-selection. High genetic differentiation was observed between populations (*R*_ST_ = 0.353) and haplotypes (*R*_ST_ = 0.133 to 0.418), indicating limited gene flow and probable local adaptation. Principal coordinates analysis and the neighbor-joining phylogenetic tree clearly distinguished the three haplotypes. Among-populations phenotypic difference (*P_S_*_T_) was lower than overall *R*_ST_ for plant height, SD, and fresh and dry mass of flowers and seeds, whereas *P*_ST_ estimates for LL and LW exceeded among-populations *R*_ST_, suggesting divergent selection, while local adaptation might have occurred in IL, LL, and flower masses. Genetic drift probably influenced among-populations IL differences.

## 1. Introduction

The spread of invasive alien species (IAS) has received a great deal of attention, given the adverse ecological and economic effects that they can impose on invaded ecosystems [1,2]. Why invasive species expand their range faster in non-native regions, but not in their native range, and how these species become dominant components of non-native habitats while being minor constituents of their native ecosystems [3] are still open questions. Alien plants are those plants that have been introduced to an area from their native range, either accidentally or intentionally, whereas invasive plants are those plants that are non-native to an ecosystem and which may cause a threat to native biodiversity and ecosystem integrity in many ecologically sensitive parts of the world. Very recently, the International Union for Conservation of Nature has come up with a standard Environmental Impact Classification of Alien Taxa (EICAT), based on the magnitude of the detrimental environmental impacts. Earlier studies have indicated that invasive species often possess specific demographic or physiological traits, such as faster growth rates, greater productivity, high fecundity, greater tolerance to environmental extreme events, or very effective dispersal compared to native and non-invasive introduced species [4,5,6,7]. These traits enable introduced species to spread very extensively and achieve high abundance. The origin of such variations in traits that influence success in competition between invasive and native species under natural conditions may include geographic isolation, genetic drift, divergent selection, phenotypic plasticity [8], and rapid adaptive evolution [9,10]. Whether phenotypic divergence among and between populations of alien species is due to one or more of these factors is a debate that remains to be resolved. 

The phenotypic differences within and among populations of invasive species in their native and non-native ranges are widely speculated [11,12]. The colonization of new areas by invasive species would be associated theoretically with founder diversity [13], phenotypic plasticity, and environmental conditions at the points of origin [14], various ecological and evolutionary processes associated with geographical zone [15], and population bottlenecks that reduce within-population genetic diversity and increase genetic differentiation among populations [16]. These population genetic differentiations have important effects on species invasiveness, which is apparently linked to increased genetic variation and evolutionary potential [17]. Moreover, population genetic analyses can increase our understanding of the origin and expansion of invasive species and invasive populations [18,19], or haplotypes (i.e., individuals with a specific group of alleles that are inherited within a species), and the same could have useful management implications as well. Previous research has typically focused on detecting how invasive haplotypes have invaded and replaced native haplotypes [20,21]. This replacement is most often due to better performance of invasive haplotypes compared to native haplotypes with respect to morphological traits, such as plant height and size, and to reproductive success. However, little attention has been paid to understanding the variation patterns of such invasive haplotypes in their native regions. Further, the causes of morphological differences between invasive and natural populations of IAS are not well understood. Thus, investigating phenotypic and genotypic differentiation between invasive and native haplotypes in wild populations should contribute to the understanding of mechanisms behind the invasion of alien species. 

Among-population phenotypic differentiation in the wild is effectively estimated using *P*_ST_ (i.e., the phenotypic analogue of *Q*_ST_); [22,23,24]. Lande (1992) [25] stated that genetic variance in phenotypic traits between populations is expected to equal that of variation in neutral molecular loci under the influence of the forces that drive evolution. Indeed, the level of differentiation of quantitative traits should be similar to the level of differentiation at neutral loci, as divergent selection in quantitative traits induces more morphological differences than would be expected for neutral molecular markers [24]. For this reason, a valuable method for investigating morphological differences between invasive and natural populations of IAS is to compare divergence in allele frequencies to divergence in quantitative traits. Whether selection or genetic drift is responsible for phenotypic divergence is a complex task and requires the initial step of comparing genetic differentiation with phenotypic differentiation. Phenotypic differences between populations are often assumed to be adaptive, and epigenetics are reported to play key role on adaptation of invasive species [26]. Both molecular and quantitative variations can be influenced by non-adaptive processes that involve random genetic drift and gene flow [27,28]. Under these conditions, it would be especially challenging and interesting to determine whether phenotypic differences between populations represent the outcomes of adaptive or non-adaptive processes.

Common reed (*Phragmites australis* [Cav.] Trin. ex Steud; Poaceae) is a polyploid perennial grass species with clonal architecture [29,30,31]. The species is distributed worldwide and is highly invasive in Kashmir Himalayan aquatic habitats [32]. In Kashmir, India, this species was earlier reported as a South American native [33] and European native [32], but substantial molecular evidence for the nativity of this species is lacking. Saltonstall (2002) [29] sampled individuals of *P. australis* worldwide and recovered 27 haplotypes of this species on the basis of the sequences of two chloroplast DNA (hereafter, cpDNA) fragments. In North America, both native and exotic haplotypes of the species are found. Introduction of an exotic genotype of *P. australis* (Haplotype M) and its rapid spread, aided by anthropogenic disturbance, has contributed significantly to its invasion and range expansion in North America [29]. Recently, Guo et al. [34] showed that evolutionary mechanisms act differently in the native and introduced ranges of *P. australis* and that invasive populations of European origin have evolved to adapt to a different climate and to human-made habitats in North America. In Quebec, Canada, the native haplotype is characterized by a red stem and ligule base (i.e., the junction of the leaf blade with the leaf sheath) with fewer hairs, compared to the green stem of invasive haplotypes, which have more hairs on the ligule. Moreover, the inflorescence of the native haplotype is less dense relative to that of the invasive haplotype, containing fewer seeds [35,36]. The invasive haplotype in Quebec tends to grow preferentially along roads and in other human-made habitats, while the native haplotype prefers undisturbed, pristine habitats [37,38,39,40]. In India, the invasive *P. australis* haplotype is found to occur widely in various aquatic habitats, especially in Kashmir Himalayan wetlands. 

The objectives of this study were: (i) to assess and compare the phenotypic and genetic diversity of invasive populations of *P. australis* in Kashmir and Quebec, together with native haplotypes of this species in Quebec, and (ii) to assess evolutionary similarities between them.

## 2. Results

Restriction fragment length polymorphism (hereafter, RFLP) analysis confirmed that there was only one native population (QN1) in our sampled populations, instead of five as thought based on morphological variations. In utilizing the cpDNA PCR-RFLP analyses, we found that the Quebec population QN1 shared the same restriction pattern as native populations in USA [41]. Further, we found that ten populations (QE1 to QE12) exhibited the same restriction sites as haplotype M [42]. Results for the sequence *trnT*-*trnL* in the native Quebec QN1 population was 99% similar to haplotype E2 (NCBI accession no. AY016325; [42]). Sequence *trnT*-*trnL* in the invasive Quebec QE1 population and sequence *trnT*-*trnL* in the Kashmir KE1 population were 99% similar to haplotype 4 (NCBI accession no. AY016327; [42]). BLAST analysis was done in GenBank NCBI database for the *rbcL*-*psa*I intergenic spacer, and for native Quebec QN1, the sequence was identical to haplotype E2 (100% identity with NCBI accession no. JQ409547; Freeland and Vachon Unpublished data). Sequence *rbcL*-*psa*I in the invasive Quebec QE1 population was 99% similar to haplotype 4, while Kashmir KE1 population was identical (100% similar) to the same sequence of haplotype 4 (NCBI accession no. AY016335, [42]). All nine populations from Kashmir displayed the same restriction pattern as haplotype M. Based on this information, we considered the putative native Quebec populations of Lac St-Louis, Parc Louis-Racine, Sablière Colette, and La Pocatière as invasive *P. australis* populations, referring to them instead as QE9, QE10, QE11, and QE12.

### 2.1. Phenotypic Diversity

The invasive *P. australis* plants of the Ange-Gardien population, Quebec (QE4) were the tallest (Mean ± SE: 230.74 ± 55.54 cm height), followed by the invasive haplotypes of Bernière, Quebec (QE8: 226.72 ± 34.63 cm height), and the invasive haplotypes of Eastman, Quebec (QE6: 212.44 ± 68.96 cm height; Table 1). The thickest plant stems were recorded for invasive *P. australis* plants from Kuhunus Wullar populations (KE5: 7.83 ± 1.362 cm), Ashaibagh (KE2: 7.78 ± 1.332 cm) and Rangharstop (KE4: 7.74 ± 1.264 cm; Table 5). The heaviest flowers in terms of fresh and dry mass were also recorded in Ashaibagh, Ange-Gardien, and Bernière populations (Table 1). The highest intermodal length was recorded in plants from Bernière (QE8: 18.00 ± 3.749 cm), La Pocatière (QE1: 16.16 ± 2.407 cm), and Princeville (QE7: 16.77 ± 2.266 cm; Table 1). The longest leaves were recorded in Ashaibagh, Rangharstop, Ange-Gardien, and Eastmann *P. australis* populations, whereas the widest leaves were recorded in invasive *P. australis* plants from Kashmir (Table 1).

Highly significant variation was observed among plants, whereas significant to highly significant variation was found among populations for the measured characters, except for leaf width (Table 2). The variation between individual plants and between populations most often contributed to the bulk of total variation in the measured characters, except for leaf width (Table 2). Additionally, among-populations variation was higher than between-haplotypes variation for all the characters that were measured (Table 2). While *P. australis* plants from Kashmir had more highly significant (*p* < 0.001) heavier flower dry mass (3.59 ± 0.59 g) than did Quebec plants (2.55 ± 0.116 g), invasive *P. australis* plants had significantly wider leaves (2.04 ± 0.036 cm) than did native plants (1.65 ± 0.05 cm). Additionally, except for flower and dry seeds mass, no variation was found among regions for all measured characters (Table 2).

### 2.2. Characterization of Phenotypes

**Figure 1:**
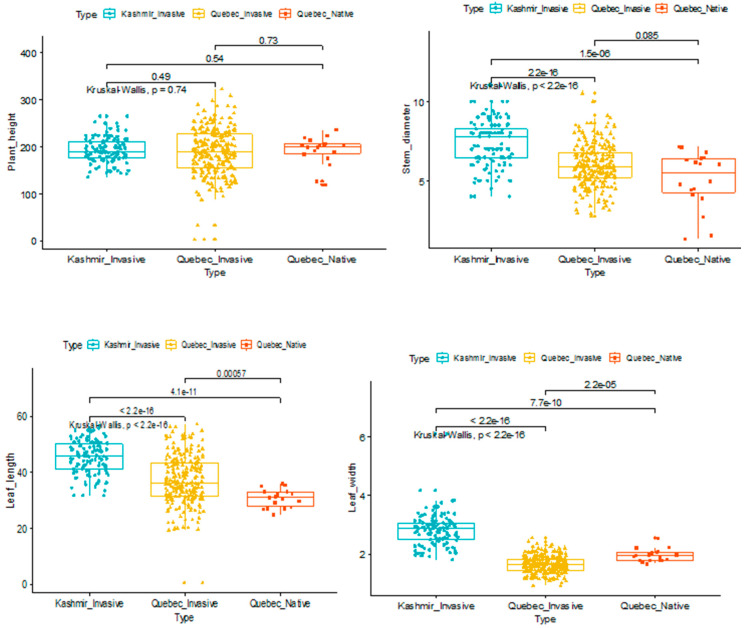
Biplot analysis of the studied 16 groups of *Phragmites australis* plants from three environments (Kashmir invasive, Quebec invasive, and Quebec native) based on seven morphological traits.

To investigate the effects of morphological traits in distinguishing the haplotypes that were investigated, we analyzed the distribution of each trait among haplotypes. Though stem diameter, plant height, leaf length, and width distinguished the haplotypes that were studied, the differences were not significant (Figure 1). 

**Figure 2:**
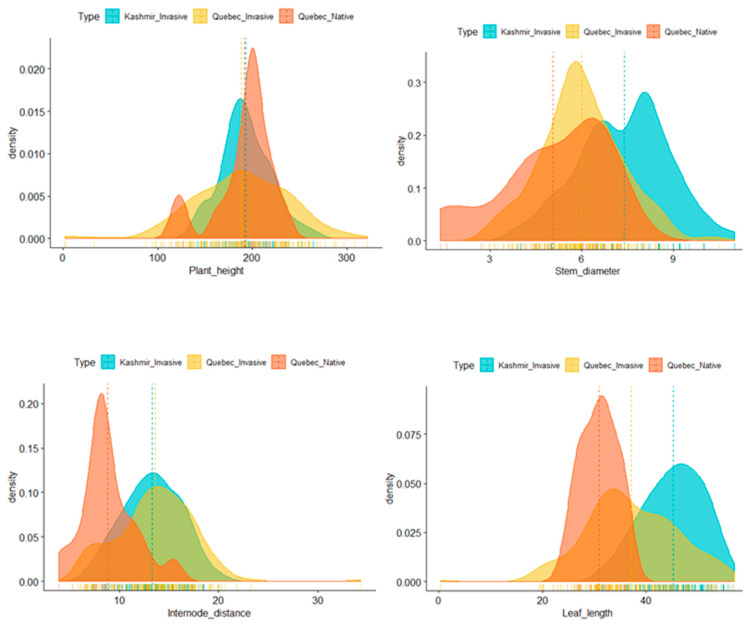
Distribution of phenotypes for the plant height (**upper left**), stem diameter (**upper right**), leaf length (**bottom left**), and leaf width (**bottom right**) are represented by histogram based on the average phenotype value of each *Phragmites* plants across different environments.

To explore descriptors, we measured seven phenotypes (plant height, stem diameter, fresh flowers, dry flowers, internode length, and leaf length and width) in *P. australis*. A bimodal distribution was observed for stem diameter, internode length, leaf length, and width (Figure 2). Normal distributions without any significant skewness were observed for the rest of traits.

**Figure 3:**
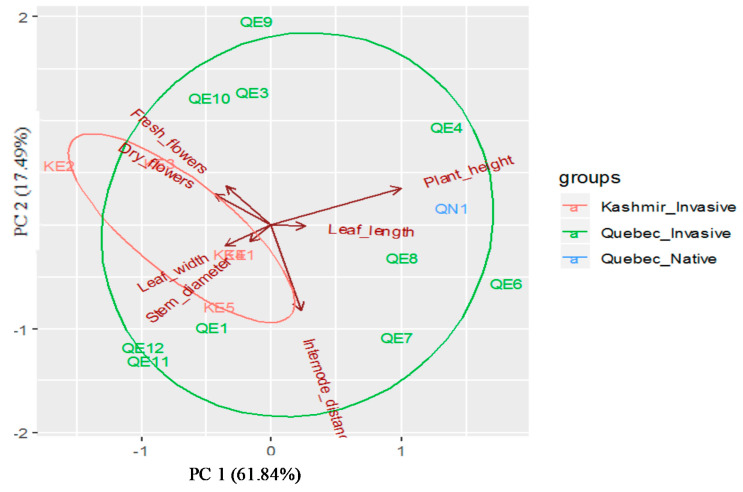
Principal component analysis plot showing different haplotypes for 378 *Phragmites* plants based of seven phenotypic traits.

Principal component analysis (PCA) indicated two principal components with eigen-values greater than 1. The PCA grouped the seven *P. australis* descriptors into various components, with the first two explaining 79.33% of the variation, while the first principal axis (PC1) alone explained 61.84% of the variation (Figure 3). The PC1 had high loading for leaf width. The second axis (PC2), explaining about 17.49% of the total variation, was correlated with plant height, stem diameter, fresh flowers and seeds, dry flowers, and seeds, internode length, and leaf length, indicating the usefulness of descriptors in *P. australis* (Table 3).

### 2.3. Phenotypic Differences and Heritability

Moderate *P*_ST_ estimates were observed among populations, ranging from 0.12 for flower and seed fresh mass to 0.41 for leaf length (Table 2). The measured traits were weakly (leaf width) to moderately (internode length, leaf length, and flower masses) heritable (Table 2). 

### 2.4. Genetic Diversity 

The polymorphism information content (PIC) varied from 0.4 (PaGT11) to 0.9, with an average of 0.8 (Table 4). Based on the number of alleles, it was found that *P. australis* in Kashmir shows hexaploidy as the number of alleles from each population ranged from 1–6; for Quebec populations, it shows tetraploidy as the number of alleles ranged from 1–4. However, Quebec native population plants seem to be diploid, having only 1–2 alleles (Table 4). All loci that were surveyed were variable in each population. Within-population genetic diversity indices are summarized in Table 5. The mean number of alleles per locus ranged from 1.89 to 4.56, and the overall mean number of alleles per locus amounted to 8.56. The mean number of alleles per polymorphic locus ranged from 1.61 to 3.37, whereas the overall mean number of alleles per polymorphic locus amounted to 4.16 (Table 5). For *P. australis* populations in Kashmir, *A* ranged from 2.00 to 4.56 and *A*_p_ ranged from 2.04 to 3.37. The range of values for *A* and *A*_p_ was not as wide in the Quebec populations, and respectively, 1.89 to 3.56, and 1.61 to 2.32. Mean percentages of polymorphic loci ranged from 66.67% (KE9, Ganderbal, Kashmir) to 100% (KE1, KE4, KE5, KE6, KE7, and KE8) for Kashmir populations. For the Quebec populations, *P* ranged from 88.89% (QE1, QE3, QE6, QE8, QE9, QE12, and QN1) to 100% (QE4, QE7, QE10, and QE11). The overall percentage of polymorphic loci was 92.78% for all study populations. Allelic richness for Kashmiri and Quebec populations ranged from 1.81 to 2.54, and 1.54 to 2.18, respectively. Observed and expected heterozygosities for Kashmir populations were 0.70 and 0.54, respectively; for Quebec populations, *H*_O_ was 0.38 and *H*_E_ was 0.39. Overall mean observed and expected estimates of heterozygosity were 0.52 and 0.46, respectively, while *H*_O_ and *H*_E_ per population ranged from 0.23 (QN1; Lac St François, Quebec) to 0.78 (KE6; Saderkote wullar, Kashmir) and from 0.20 (QN) to 0.66 (KE6), respectively (Table 5). 

### 2.5. Population Genetic Structure

**Figure 4:**
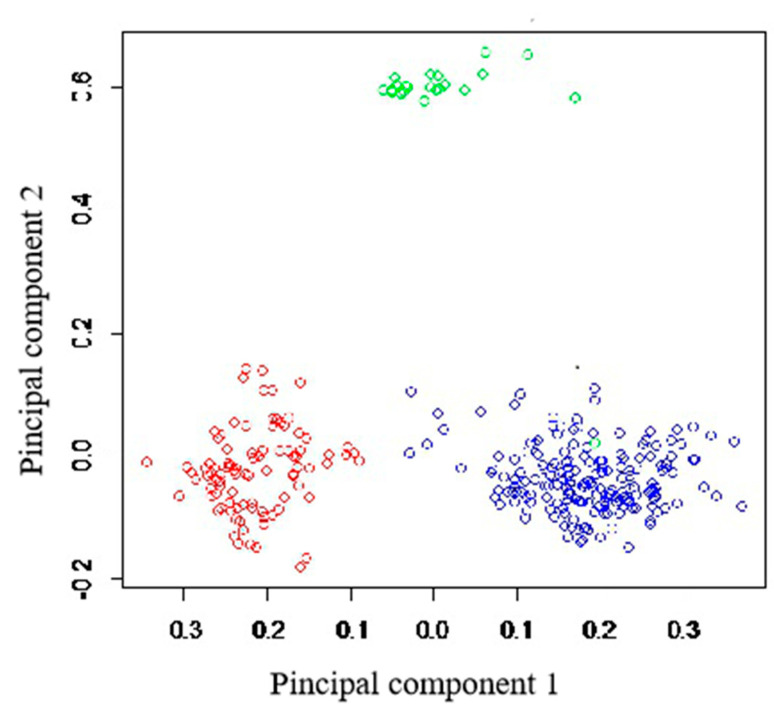
Principal coordinate analysis with Bruvo distances, based on molecular data displaying population structure of Kashmir and Quebec invasives and Quebec native *Phragmites* between principal component analysis (PCA) 1 and PCA 2 (Blue—Quebec invasive populations; Green—Quebec native population; Red—Kashmir invasive populations).

High genetic differentiation was observed among populations (overall *R*_ST_ = 0.353), ranging from 0.133 between invasive populations from Kashmir and Quebec, to 0.418 between Quebec native and invasive populations. These differences were best illustrated by the results of principal coordinate analysis (Figure 4). The *P. australis* plants separated into three groups: Kashmir invasive, Quebec natives, and Quebec invasive, which each formed one group (Figure 4).

**Figure 5:**
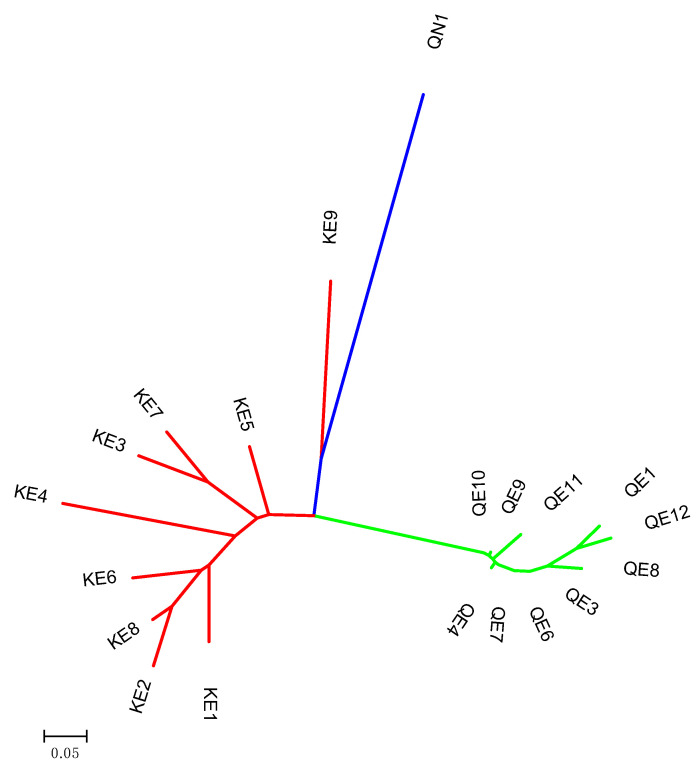
Phylogenetic tree of *Phragmites australis* populations in native and invasive ranges based on SSR markers using the neighbor-joining method. Colors on branches classified subpopulations, mainly differentiated by geographical origin and ranges.

The phylogenetic tree (Figure 5) was congruent with principal coordinate analysis results (Figure 4), as the populations of native and non-native *P. australis* that were studied were found to be very structured. Clearly, ten populations belonging to Quebec invasive (QE1, QE2, QE4, QE6, QE7, QE8, QE9, QE10, QE11, and QE12) were grouped together, completely distant from the population of Quebec native (QN1; Figure 5). Kashmir invasive populations were clustered in the same group, whereas KE9 were close to QN1 population following by a sub-group containing height populations (KE1, KE2, KE3, KE4, KE5, KE6, KE7, and KE8; Figure 5). 

### 2.6. R_ST_- P_ST_ Comparisons

Among-population phenotypic differentiation estimates for plant height (0.23), plant stem diameter (0.25), and fresh and dry mass of flowers (0.12 and 0.14, respectively) were less than overall *R*_ST_ (0.35), whereas *P*_ST_ estimate for leaf width (0.39) and leaf length (0.41) exceeded the genetic differentiation at nine cpDNA microsatellite loci among the *P. australis* populations that were surveyed in the present study. *P*_ST_ estimate for internode length (0.33) was comparable with among-population *R*_ST_ estimate (0.35).

### 2.7. Relationships between Genetic and Geographic Distances

All the populations from both regions revealed a weak positive relationship between genetic distances and environmental variables (r^2^ = 0.389, *p* < 0.001). When Mantel test was calculated within each region it again showed a weak but significant correlation between the two variables in Quebec (r^2^ = 0.158, *p* ≤ 0.05) and non-significant correlation in Kashmir (r^2^ = 8.06 × 10^−3^, *p* = 0.5570).

## 3. Discussion

The behavior of *P. australis* in various lakes, wetlands, and other water bodies of Kashmir is that of a typical invader in terms of forming the monotypic stands, attaining great abundance, and outcompeting native species [43,44]. Previous studies that were carried out in 2018 [45] based on field observations reported this species at the stage V (widespread and dominant) on the stage-based hierarchical model (CM model) propounded by Colautti and MacIsaac [46]. Similarly, earlier studies conducted in 2007 [33] reported this species as a South American native and Shah et al. [32] in 2014 reported it as a European native based on published, web-based sources as well as phytogeographical distribution of this species, but substantial molecular evidence for the nativity of this species is lacking. Based on the above evidence, many authors declared that *P. australis* was introduced in India, especially in Kashmir Himalayan region [44,45]. There is hardly any study conducted in Kashmir, India, or elsewhere, that suggests native range of *P. australis* in this part (Kashmir Himalaya) of Asia. The present study aimed at increasing our understanding of the phenotypic and genetic diversity of invasive populations of *P. australis* in Kashmir and Quebec. Only one native *P. australis* population in Quebec was identified during field surveys on reported natural distribution of the species across Quebec province and all the populations from Kashmir turned out to be invasive as revealed by cpDNA PCR-RFLP analyses. Such findings indicate that invasive haplotypes are becoming more dominant in the natural distribution range of the species in the sites of study. This is the first study in India, especially in Kashmir Himalaya, that provides molecular evidence regarding the invasive status of *P. australis* in this region. Such successful invasion of alien haplotypes in their non-native range is due to the fact that invaders usually perform better than natives in terms of life-history traits such as mature plant size and fecundity, thereby reducing the size of, or even replacing, native populations [4,7]. Further studies in this direction need to be carried out by taking samples from a larger global range, including native European populations of *P. australis*, in order to provide greater understanding of the origin and phylogeography of this species.

### 3.1. Phenotypic Variation

Given the premise that the invasive populations perform better than native ones in terms of various growth-related traits, we found the highest values of seven growth-related traits of *P. australis* within invasive populations. These results are congruent with that of other studies [47,48,49], although DeVrie et al. [50] indicated that morphological characters cannot discriminate reliably between invasive and native *P. australis* individuals in North America. Further, local environment highly affects phenotypic traits in common reed [51], explaining the difficulty in differentiating invasive and native individuals from these traits. However, plasticity in common reed is trait-specific [51,52]. Similarly, Pysek et al. [53] compared the functional traits of 89 populations in a common garden experiment belonging to distinct clones of different origin: North America, Europe, Australia (two groups including native and invasive populations introduced from Europe), South Africa, and Far East. In general, both native and invasive populations with origins in Europe showed highest performance-related traits than other groups. North American native populations performed more poorly than both European-related groups and were similar in this respect to Australian and South African populations [53]. Invasive European populations in North America do suppress the native North American populations wherever growing in direct competition [54]. The weaker performance of Australian and South African populations in the Northern Hemisphere is due to weak genetic differences or environmental similarities [53].

Plant height plays an important role in invasiveness, as Crawley et al. [55] and Canavan et al. [56] found that tall-statured grasses harbor a useful functional trait for invasion success. In their review, Mozdzer et al. [57] clearly indicated that introduced and native *P. australis* lineages differ both physiologically and morphologically. Introduced plants are generally taller and occur in greater densities, which results in greater productivity in the introduced lineage in nearly every study.

In the present study, plant stem diameter appeared to be linked to invasiveness of *P. australis,* given that the highest mean values were recorded for invasive populations. Yet Muth and Pigliucci [58], in comparing six growth-related traits, found very little variation among introduced invasive and introduced non-invasive plant species within two genera of Asteraceae (*Crepis* and *Centaurea*), with respect to stem diameter. As our study included one native population, we suggest that any assumptions need to be confirmed with fine-scale studies over a broader distribution range of *P. australis*, taking into account an even larger number of populations. 

Abundant flowering may also play an important role in reproductive success, which obviously influences propagule pressure and invasiveness. Our results that invasive plants in both regions significantly produce more flowers and seeds than native plants are quite striking. These traits can be taken as the indicator/s of plant invasiveness. In a survey of 46 native and 45 introduced populations of *Solidago gigantea*, Jakobs et al. [59] found that total plant biomass was larger for invasive than native plants. Some genetic studies have shown that abundant seed production and seed-based recruitment are more common in *P. australis* compared to dispersal by stolons and rhizomes [60,61,62]. Albert et al. [63], however, attributed the success of this invader in North America to a reproduction strategy combining both the advantages of generative and vegetative propagations.

Comparing the leaf characteristics of invasive and native populations showed that the former exhibit higher values for leaf area than the latter. Bimodal distribution observed for traits related to stem diameter, internode distance, leaf length, and width suggest that a major gene controls those four important characters related to leaf and stem sizes in *P. australis*. Although there was not significant among-population variation in leaf width, results from the present study revealed maximum leaf length and leaf width for invasive populations, while the lowest values were encountered in native population. Furthermore, variation in leaf width was very highly significant between haplotypes in the present study, with invaders appearing to perform better with respect to this quantitative trait as well. Successful invaders are characterized by a wider range of leaf areas and by greater phenotypic plasticity [64]. Several studies have shown that invasive species have higher resource capture-related traits and possess more efficient photosynthetic machinery compared to native or non-invasive species [11,65,66,67]. Invasiveness is strongly related to leaf traits that are associated with rapid C capture and net CO_2_ assimilation [68,69] and relative growth rate [70]. 

### 3.2. Characterization of Phenotypes

The characterization of among-haplotype variation could provide clues on co-selection of traits in case hitchhiking occurs [71]. Indeed, a group of characters may distinguish closely related taxa, which are geographically distant, because of genetic drift or hitchhiking. Similar effects are also observed when a major gene controls the characters of interest. Results of the present study revealed that at least stem diameter, leaf length, and width may undergo co-selection or the traits are under the control of the major gene. Genome-wide association studies in the species may provide more insights on the issue. 

### 3.3. Genotypic Studies

After obtaining useful insights into phenotypic trait-based variations in native and invasive populations’ vis-à-vis invasiveness in *P. australis*, our objective was to confirm such variations at molecular level. Results from the present study indicate that in general, highest levels of genetic diversity are found in invasive populations, while the lowest levels of diversity are recorded in native population, consistent with [72]. The highest levels of genetic diversity in invasive populations may explain adaptability of invaders in non-native habitats [9,10]. The low levels of genetic diversity in the only one native population that was identified in the present study are congruent with the results of [73], which observed lower levels of genetic diversity in native *P. australis* populations than in invaders at many of cpDNA loci. This is contrary to what was observed by Zhao et al. [74], who reported highest genetic diversity among native Mexican populations of *Solanum rostratum* Dunal and significantly reduced diversity in invasive populations within the USA and China, using ten microsatellite markers. Since cpDNA PCR-RFLP haplotype identification analyses in the present study revealed only one native population, instead of five as thought on the basis of morphological variations, and all the other populations are exotic, it seems that invasion has either replaced the native populations or intraspecific hybridization might have occurred between native and introduced lineages of a species that has increased invasiveness and lead to the decline of native lineages [54]. We suggest that a broad-scale study including more continents than what has been done in our study, after identifying more native populations, be carried out to confirm or refute our findings.

Being a cryptic invader, *P. australis* has emerged as an important model system for understanding mechanisms of invasion. There have been few population-level studies to directly evaluate differences in genetic diversity, reproductive mode, and dispersal between native and non-native *P. australis*, particularly in western North America [62]. In view of the lack of any population genetics studies on *P. australis* in India, we have attempted here to assess its genetic diversity through allelic and population variation of microsatellite loci. It is noteworthy that the mean number of alleles per locus in the present study (*A* = 8.56) was comparable to the findings of Saltonstall [73] on European *P. australis* populations (*A* = 8.0), although it was greater than the average (*A* = 7.6) as reported by Paul et al. [75] on *P. australis* in the United Kingdom. In the present study, we most frequently came across tetraploidy and hexaploidy among regional groupings of invasive populations of *P. australis* that were collected across Quebec and Kashmir, although the native population in Quebec seems to be diploid. Hence, it appears that polyploidy might have played some role in invasiveness of this species. Clevering and Lissner [76] found that tetraploidy was the most common ploidy of *P. australis* in Europe and North America, while octaploids (8x) were predominated in Asia. Such results suggest that invasive plants are more likely to be polyploids, while native plants are more likely to be diploids as was reported in some other studies [77,78]. Pandit et al. [79] showed that invasive plant species are generally found to have high chromosome counts and to be polyploids, in contrast to rare species that are diploids and have lower ploidy ratios. In a related study, it was shown that the highly invasive plant species in Singapore were all polyploids [80], thereby indicating that polyploidy contributes to invasion success. Interestingly, invasive Kashmir and invasive Quebec populations of *P. australis* were more similar to one another, both in terms of the number of alleles and fragment size, than to the native Quebec population that showed unique numbers of alleles and fragment sizes. With an increased native population size, more differences might be seen. However, Saltonstall (2003a) [30] reported the same allelic variation among native *P. australis*, while highest allelic diversity was observed among introduced *P. australis* that were collected in North America.

Our results indicate that *P. australis* populations that were studied are structured, which is in agreement with Guo et al. [34]. While the latter study revealed the presence of substructures to delineate the population structure in common reed, Guo et al. [81] postulated that the climatic niche had shifted between the native and introduced populations of the invasive European lineage. The authors reported strong effects of geography and environment on the genetic structure of populations in the native range, but these effects were counteracted by human-related factors manifested through colonization of anthropogenic habitats in the introduced range. Another possible explanation of the substructures observed in our study may be borrowed from Albert et al. [63], who while investigating the reproduction of *P. australis* in eastern North America proposed that long-distance seed dispersal is imperative for common reed along roadsides and in marshes; whereas, plant fragments as well as seeds were shown to be important for dispersal to short distances along roads at least in the areas of its abundance. Further, robust reproductive strategy, both through sexual and asexual modes, was shown to mainly contribute to the invasive success of this species in North America. Our phylogenetic analysis easily identified three mains groups, with each group showing clear specificity in terms of the investigated traits.

### 3.4. Phenotypic vs. Genotypic Differentiation

Our results suggest that environmental conditions in Quebec and Kashmir are not as much contrasting to induce divergent selection on quantitative traits through strong local population pressures [82]. This is because there was no major variation in genetic structure between Quebec and Kashmir *P. australis* populations, and no significant phenotypic variation was recorded between Quebec and Kashmir populations, except for flower and seed dry mass. Such non-spatial geographic differences in phenotypic traits are not common and may be caused by probable multiple introductions [17,83]. However, difference in flower and seed dry mass between the regions indicates that there might be differential effect of selection on quantitative characters.

The among-population phenotypic divergence that was observed in this study regardless of region is not surprising and may be explained by several factors, including plasticity, genetic drift, and divergent selection. As *R*_ST_ differed to *P*_ST_ for six out of seven studied characters, divergent selection appears as the most probable cause of among-populations difference in quantitative traits [84]. No apparent physical barrier to gene flow existed between sampled populations within the region, suggesting that genetic barriers such as different ploidy levels or the high rate of local lateral vegetative propagation of the species may explain the reduced gene flow among populations. Results from the present study are in contrast with that of [30], which investigated patterns of differentiation in nine microsatellite loci in *P. australis*. Whether the observed phenotypic divergence in *P. australis* populations is heritable is not known. Investigating fine-scale local spatial invasions (i.e., within geographic regions) would certainly shed more light on the possible contributions of plasticity to patterns of among-population phenotypic variation in *P. australis*.

The *R*_ST_-*P*_ST_ contrast is useful for evaluating the relative contributions of non-adaptive and adaptive processes in shaping quantitative traits. Local adaptation and evolutionary potential have been advocated as greatly influencing the outcomes of invasion by alien species [10,17]. Heritability estimates of the characters that were studied may provide clues on local adaptation. Results of the present study indicate local adaptation of invasive haplotypes for internode length, leaf length, and flower masses, indicating that these traits may influence invasion success in the species. On the other hand, isolation by distance or divergent selection are the main hypotheses that explain phenotypic divergence in nature within species [8]. As we observed a contrast between *R*_ST_ and *P*_ST_ estimates for six out of the seven traits that were investigated, we tested the correlation between genetic distances and geographical distances. Our results showed no or weak relationships between genetic and geographic distances in the regions that were surveyed. Thus, the hypothesis that could explain the phenotypic divergence observed between populations in traits that were investigated in our study is divergent selection for plant height, plant stem diameter, fresh and dry flower mass, leaf length, and leaf width coupled with local adaptation in internode length, leaf length, and flower masses. We also postulate that internode length variation between populations are explained by genetic drift. However, a large fine-scale study would confirm these findings.

## 4. Materials and Methods

### 4.1. Study Area and Target Species

**Figure 6:**
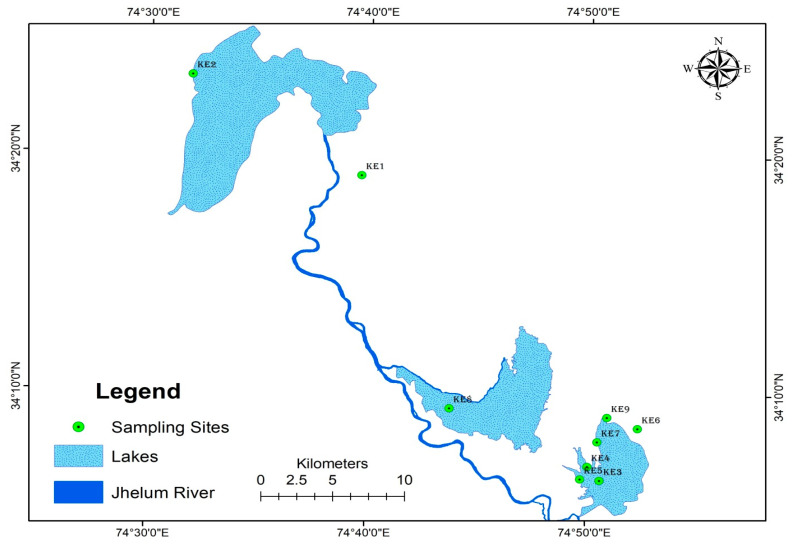
Map of the study area showing the distribution of sampling sites in different aquatic habitats of Kashmir Himalaya.

**Figure 7:**
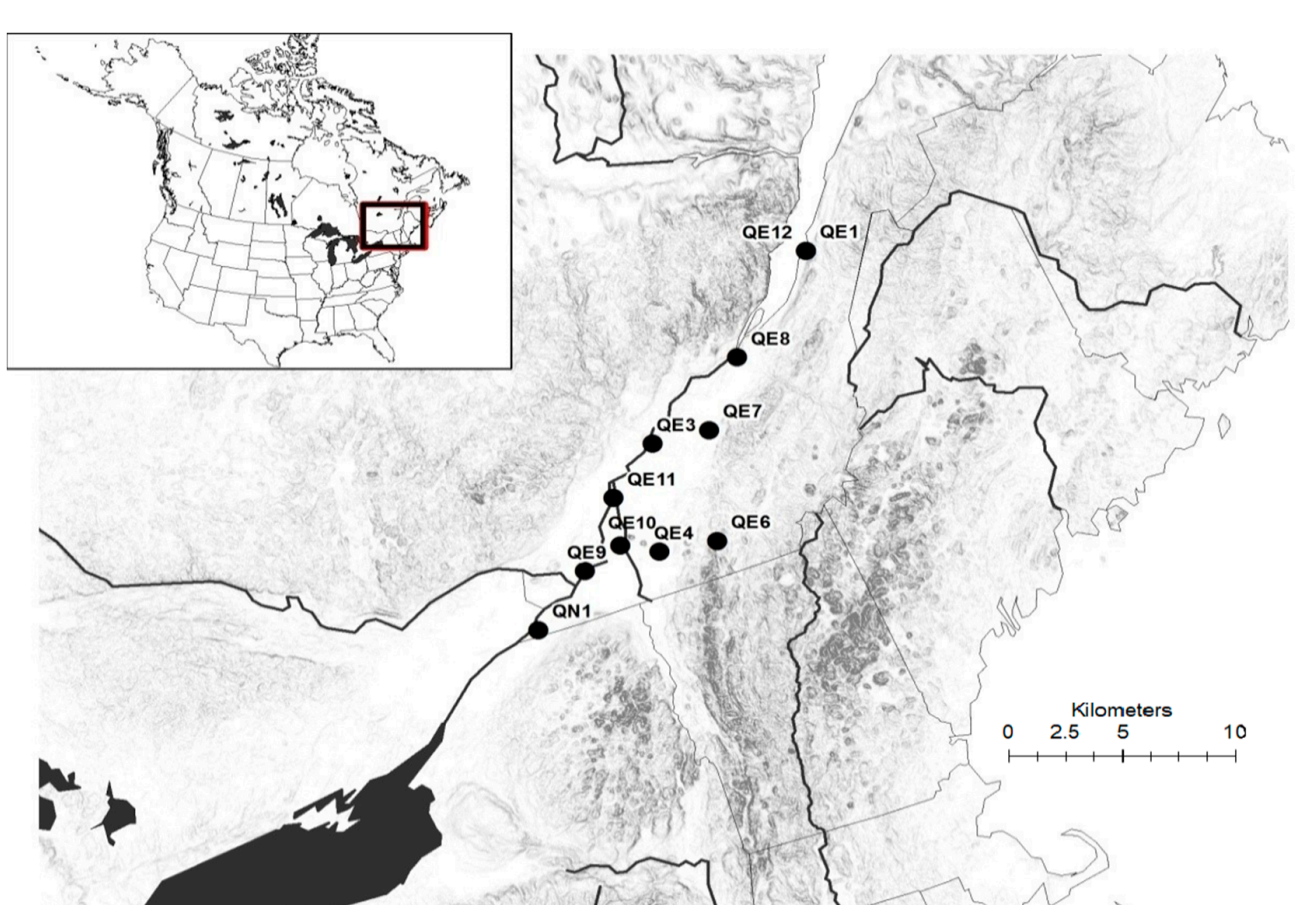
Map of the study area showing the distribution of sampling sites in different habitats of Quebec, Canada.

The target species for the present study was *P. australis*. We specifically studied wild, alien invasive *P. australis* populations in the province of Quebec, Canada and Kashmir Himalaya, India (Figure 6 and Figure 7), in addition to five putative native populations of this species in Quebec that were assumed to be native. Information about nativity and distributional range of the species was obtained from web-based sources, such as the Germplasm Resource Information Network (GRIN; http://www.ars-grin.gov/), the United States Department of Agriculture (USDA Agricultural Research Service 2015 http://plants.usda.gov/ [85]), and the Integrated Taxonomic Information System (USDA Germplasm Resources Information Network https://doi.org/10.15482/USDA.ADC/1212393 [86]). However, information about the native populations in Quebec were obtained from the specimens of the target species that were stored in the two main herbaria of Quebec, viz., Louis-Marie Herbarium (QFA, Université Laval) and Marie-Victorin Herbarium (MT, Institut de recherche en biologie végétale, Université de Montréal), together with those held in the Kashmir University Herbarium (KASH), and the list of native populations provided by Lavoie et al. [87] onwards were also consulted.

### 4.2. Sampling Procedure 

A total of 16 populations (Table 6) were investigated in Kashmir and Quebec for morphological traits, including five populations that were invasive to Kashmir; six putative populations that were invasive to Quebec, and five putative native populations in Quebec on the basis of morphometric traits [88]. Twenty-five individual plants, which were separated by at least 10 m from one another to increase the likelihood that they were distinct genets [62], were collected in each population. Morphological traits that were targeted in the present study included stem diameter, plant height, leaf length, leaf width, and internode length (Table 7). The status of populations (invasive or native) was recorded in the field based on morphological differences between plants [88].

A total of 20 populations were sampled in this study for genetic study, including nine populations from Kashmir and eleven populations from Quebec (Table 6). We designated only one population out of 20 populations as native, using cpDNA PCR-RFLP analysis. Collection sites in each region were separated by distances of 10–100 km. At each site, we collected one population, and, within each population, 25 individual plants were collected for molecular analysis. Fresh leaf samples (each about 1 cm^2^ in area) were collected in tubes with desiccant and kept frozen at −20 °C prior to DNA extraction. Genomic DNA from plant species was extracted using the CTAB method [89].

### 4.3. Genotyping

To verify whether *P. australis* individuals belonged to native or to introduced genotypes, a cpDNA PCR-RFLP analysis that had been developed by Saltonstall [41] was used. Briefly, two non-coding regions of the chloroplast genome [*trnT* (UGU)-*trnL* (UAA) and *rbcL*-*psa*I] were amplified using the primers described by Saltonstall [41]. PCR products were digested using the restriction enzyme *Rsa*I for the *trnL*b region and *Hha*I for the *rbcL* region following the manufacturer’s protocol (Promega, Madison, WI). Restriction fragments were electrophoresed in ethidium bromide-stained 3% TAE agarose gels and visualized using UV light. We also searched for matches with a Blast tool provided by NCBI. To confirm the results, PCR products were sequenced on an ABI 16-capillary genetic analyzer 3130XL (Applied Biosystems, Foster City, CA, USA). Sequences were edited and aligned using BioEdit version 7.0.5 [90]. The BLASTn algorithm [91] was used to query GenBank (NCBI) to determine the closest *P. austalis* haplotype. Phylogenetic analyses were conducted using MEGA 7 software [92], and the Neighbour-Joining (NJ) method [93].

To assess the genetic diversity within and among populations of native and invasive *P. australis* across regions, we used a set of 9 cpDNA microsatellite loci, *viz*., PaGT-4, PaGT-8, PaGT-9, PaGT-11, PaGT-12, PaGT-13, PaGT-14, PaGT-16, and PaGT-22, which were developed by Saltonstall (Table 8) [30]. Amplifications were performed as two multiplexes and five single reactions, each in a total volume of 15 µl containing 50 ng of template DNA, 0.5 mM each of the two forward and reverse primers, 0.2mM dNTPs (Applied Biosystems, Life Technologies, New York, NY, USA), 1.5 mM MgCl_2_,1x PCR buffer (10 mMTris, pH 8.0, 50 m MKCl, and 50 mM ammonium sulphate; Sigma Aldrich, St. Louis, MO, USA), 0.5 mM Fluorochrome (Applied Biosystems, Life Technologies), and 1 unit of Taq DNA polymerase (Sigma-Aldrich). Multiplexes PCR employed an annealing temperature of 55 °C and amplified loci PaGT 4 with PaGT 14, and loci PaGT 8 with PaGT 13. Single PCR employed an annealing temperature of 55 °C for loci PaGT 9 and PaGT 12, and 56 °C for loci PaGT 11, PaGT 16, and PaGT 22. Single PCRs were subjected to the same reaction conditions as the multiplexes (Table 8).

Two PCR protocols with different annealing temperatures were used during this study (Table 8): (1) Amplification conditions were as follows: 94 °C for 3 min followed by 35 cycles of 94 °C for 30 s, 55 °C annealing for 30 s, 72 °C for 30 s, and a final extension of 72 °C for 5 min for loci PaGT 4 with PaGT 14, and loci PaGT 8 with PaGT 13, PaGT 9, and PaGT 12; (2) Amplification conditions were as follows: 94 °C for 4 min followed by 31 cycles of 94 °C for 45 s, 56 °C annealing for 2 min, 72 °C for 1 min, and a final extension of 72 °C for 2 min for loci PaGT 11, PaGT 16, and PaGT 22. Fragments were measured using an ABI PRISM 3130XL Analyzer (Applied Biosystems, Carlsbad, CA, USA) and scored using Gene mapper v4.0 software (Applied Biosystems).

### 4.4. Analysis of Phenotypic Data

Variance components were estimated in the Statistical Analysis System [94] with Proc Mixed using the restricted maximum likelihood analysis. Haplotype effects were fixed whereas region, population, and plant effects were assumed to be random, and data analyses were performed using the statistical model for a three-stage nested design [95]:

Y*_ijkl_* = μ + τ*_i_* + β*_j(k)_* + δ*_(ij)k_* + e*_(ijk)l_* (1)

where Y*_ijkl_* is the average value for the dependent variable for the *i*th region (Quebec or Kashmir), for the *j*th population nested in the *i*th region, for the *k*th haplotype (invasive or native) nested in the *j*th population and further in the *i*th region, and for the *l*th plant nested in the *k*th *P. australis* haplotype nested in the *j*th population further nested in the *i*th region; μ is the overall mean; τ*_i_*, β*_j(k)_*, δ*_(ij)k_* and e*_(ijk)l_* corresponded, respectively, to region effect (i.e., the between-region variance), to population effect (i.e., the among-population variance), to haplotype effect (i.e., the between-haplotype variance), and to plant effect (i.e., the within-population variance), the last term being the source of experimental error in the model. Post hoc multiple comparison of means was performed using Duncan Multiple Range Tests. *P*_ST_ values for each measured character were estimated using the following formula [22,96]:

*P*_ST_ = *V*_AP_/(*V*_AP_ + 2*V*_WP_) (2)

where *V*_AP_ is the among-population variance and *V*_WP_ the within-population variance.

To determine whether putative phenotypic differences are due to adaptive processes or not, an analogous of narrow-sense heritability (*h*^2^) estimate was computed as follows, as *P. australis* dispersal most often occurs through seeds than stolon and rhizomes [60,62]:

*h*^2^ = among-plant variance/total variance (3)

Similarity among different *P. australis* populations and population groups was calculated using Euclidean distance performed on standardized variables [97], while hierarchical cluster analysis between populations was carried out on distance values using the R package pvclust [98]. We characterized haplotypes and plotted the phenotypic distribution for each trait using the R ggpubr program [99]. Principal components analysis (PCA) with phenotypic traits was applied to examine the pattern of phylogeographic groups, and how close they were to each other. PCA was performed using the Facto Mine R package [100].

### 4.5. Population Genetics Analyses

Given that *P. australis* plants sampled in this study were of different ploidy levels, descriptive statistics were performed using Polysat version 1.3 [101]. These statistics included the percentage of polymorphic loci (*P*), the mean number of alleles (*A*), the mean number of alleles per polymorphic locus (*A*_p_), allelic richness (*A*_R_), and observed and expected heterozygosities (*H*_O_ and *H*_E_) for each population [102], together with unbiased estimates of population differentiation for microsatellites (*R*_ST_; [103]). Prior to analysis of molecular data, gametic disequilibrium verification between loci was performed using Fisher’s exact tests. Bruvo distances [104] were estimated between genotypes using Polysat version 1.3 and principal coordinates analysis was used to display population structure, using R package [105]. To measure the informative character of the SSR markers that were used in the present study, the polymorphism information contents (PIC) for each marker was calculated using as follows [106]:

PIC = 1 − Ʃ_i=1_^k^ P_i_^2^ (4)

where k is the total number of alleles detected per locus and P_i_ the frequency of the allele i in all populations. Genetic similarity (GS) [107] was calculated as follows:

GS = 2N_ij_/(N_i_ + N_j_) (5)

where N_ij_ is the number of fragments common to individuals i and j, and (Ni + Nj) is the total number of fragments in both individuals.

To assess the phylogenetic relationships between individuals, the distance matrix was computed as genetic distance (GD) among *P. australis* populations through the software microsatellite analyser (MSA) version 4.05, using the formula of Nei and Li [108], as follows:

GD_xy_ = 1 − (2N_xy_/N_x_ + N_y_). (6)

Where N_xy_ is the number of fragments common to individuals x and y, and (Nx + Ny) is the total number of fragments in both individuals.

Then, the phylogenetic tree was constructed using genetic distance (neighbor joining algorithm) of the 20 populations of *P. australis* using Phylip and Mega 6 software. Relationships between genetic and geographic distance were investigated using Mantel tests in the Isolation By Distance Web Service (IBDWS 3.23; [109]). Mantel Statistics was performed using 10,000 permutations to evaluate correlation between genetic and geographic distance.

## 5. Conclusions

In conclusion, it appears that invasive populations of *P. australis* exhibit better growth-related traits, especially fresh and dry mass of flowers and seeds. PCoA based on Bruvo distances clearly distinguished invasive and native populations from the sample regions. Highest levels of genetic diversity were found in invasive populations of *P. australis* in Kashmir Himalayan wetlands. The observed among-population phenotypic differences in the surveyed *P. australis* are explained by divergent selection, local adaptation, and genetic drift. Understanding the phylogeographic basis of such a diversity gradient remains an open challenge. Cluster and structure analysis delineated all populations of native and invasive *P. australis* from Quebec and Kashmir into five main groups, shedding some light on their evolutionary relationships. The important level of the genetic diversity reported in the present study should be considered in managing the native and invasive *P. australis* populations in Quebec and Kashmir. The results provide a first step for further investigations based on sampling from a larger global range, including native European populations of *P. australis*.

## Figures and Tables

**Table 1:** The summary statistics (mean and standard error (± SE)) of measured seven phenotypic traits of invasive and native species of *Phragmites australis* in 16 populations from Kashmir, India and Quebec, Canada (QN1 = native population from Quebec; QE = invasive population in Quebec; KE = invasive population in Kashmir).

PopulAtion	Plant Height (cm) ± SE	Plant Stem Diameter (mm) ± SE	Fresh Mass Flowers and Seeds (g) Per Plant ± SE	Dry Mass Flowers and Seeds (g) Per Plant ± SE	Internodal Length (cm) Per Plant ± SE	Leaf Length (cm) ± SE	Leaf Width (cm) ± SE
**QN1**	191.64 ± 30.388 ^cde^	5.06 ± 1.794 ^fg^	0.71 ± 0.594 ^h^	0.17 ± 0.102 ^f^	8.86 ± 2.69 ^h^	30.90 ± 3.453 ^fg^	1.94 ± 0.220 ^d^
**QE12**	135.20 ± 24.643 ^f^	5.27 ± 1.315 ^efg^	2.96 ± 2.44 ^fg^	1.61 ± 1.072 ^e^	12.52 ± 1.921 ^ef^	28.99 ± 6.632 ^g^	1.64 ± 0.377 ^fg^
**QE1**	176.39 ± 35.788 ^e^	6.22 ± 1.479 ^cd^	6.07 ± 2.929 ^bcd^	3.15 ± 1.443 ^bc^	16.16 ± 2.407 ^b^	33.48 ± 8.262 ^ef^	1.80 ± 0.317 ^def^
**QE3**	193.88 ± 22.643 ^cde^	5.84 ± 0.828 ^def^	6.29 ± 3.408 ^bcd^	3.49 ± 1.952 ^bc^	12.63 ± 2.383 ^ef^	28.56 ± 4.827 ^g^	1.32 ± 0.204 ^h^
**QE4**	230.74 ± 55.540 ^a^	6.73 ± 1.110 ^bc^	6.84 ± 3.155 ^abc^	3.64 ± 1.592 ^bc^	14.40 ± 1.854 ^cd^	46.80 ± 6.018 ^ab^	1.70 ± 0.257 ^efg^
**QE6**	212.44 ± 68.965 ^abc^	6.36 ± 0.970 ^cd^	3.84 ± 3.053 ^efg^	1.71 ± 1.527 ^e^	13.89 ± 2.577 ^cde^	46.52 ± 6.170 ^ab^	1.88 ± 0.212 ^de^
**QE7**	202.36 ± 37.629 ^cd^	6.29 ± 1.398 ^cd^	5.20 ± 3.428 ^cde^	2.99 ± 1.941 ^bc^	16.77 ± 2.266 ^ab^	42.50 ± 6.013 ^cd^	1.76 ± 0.235 ^def^
**QE8**	226.72 ± 34.632 ^ab^	6.85 ± 1.408 ^bc^	7.90 ± 4.116 ^ab^	3.91 ± 2.029 ^ab^	18.00 ± 3.749 ^a^	41.57 ± 4.219 ^d^	1.77 ± 0.218 ^def^
**QE9**	173.32 ± 26.353 ^e^	4.90 ± 1.034 ^g^	3.91 ± 1.981 ^efg^	1.88 ± 1.046 ^de^	7.54 ± 1.233 ^h^	34.13 ± 4.088 ^ef^	1.59 ± 0.279 ^fg^
**QE10**	176.46 ± 30.863 ^e^	6.05 ± 1.343 ^cde^	5.94 ± 4.227 ^bcd^	2.76 ± 1.933 ^cd^	10.89 ± 3.365 ^g^	34.81 ± 5.410 ^e^	1.50 ± 0.158 ^gh^
**QE11**	126.48 ± 23.901 ^f^	4.99 ± 0.937 ^g^	2.65 ± 2.049 ^g^	1.40 ± 1.111 ^e^	12.63 ± 2.383 ^ef^	28.56 ± 4.827 ^g^	1.32 ± 0.203 ^h^
**KE1**	206.56 ± 29.509 ^bcd^	7.24 ± 1.556 ^ab^	5.89 ± 2.507 ^cd^	3.40 ± 1.558 ^bc^	13.88 ± 2.850 ^cde^	41.74 ± 4.601 ^d^	3.00 ± 0.706 ^a^

Means that are followed by the same letter in columns are not significantly different using Duncan’s Multiple Range Test; native population (QN1) in bold.

**Table 2:** Variance in *Phragmites australis* morphological characters from sixteen natural stands in Québec, Canada and Kashmir, India (SE = Standard Error).

Source of Variation	Plant Height		Plant Stem Diameter		Fresh Mass of Flowers and Seeds		Dry Mass of Flowers and Seeds		Leaf Length		Internodal Length		Leaf Width	
	Variance ± SE	*p*-Value	Variance ± SE	*p*-Value	Variance ± SE	*p*-Value	Variance ± SE	*p*-Value	Variance ± SE	*p*-Value	Variance ± SE	*p*-Value	Variance ± SE	*p*-Value
**Region**	0		0.965 ± 1.458	0.2541	0.366 ± 1.092	<0.37	0.37 ± 0.01	<0.001	31.5 ± 52.66	0.2766	0		0.69 ± 0.989	0.242
**Population (Region)**	730.06 ± 304.25	0.0082	0.371 ± 0.174	0.0165	2.160 ± 1.059	0.0207	0.577 ± 0.29	<0.001	38.83 ± 5.754	0.007	6.07 ± 2.406	0.0058	0.0298 ± 0.0228	0.095
**Haplotype (Population (Region))**	20.76 ± 0.00		0		0.140 ± 0		0.051 ± 0		0.0142 ± 0		0		0.0233 ± 0.002	<0.001
**Plant (Haplotype (Population (Region)))**	1227.30 ± 91.39	<0.0001	0.555 ± 0.125	<0.001	7.846 ± 0.654	<0.001	1.743 ± 0.195	<0.0001	27.55 ± 2.34	<0.001	6.08 ± 0.518	<0.001	0	
**Residual**	1.0096		1.123 ± 0		0.932 ± 0		0.879 ± 0		1.155 ± 0		0.884 ± 0		0.1144 ± 0.0085	<0.001
***P*_ST_**	0.23		0.25		0.12		0.14		0.41		0.33		0.39	
***h*^2^**	0.094		0.184		0.685		0.481		0.278		0.466		0	

**Table 3:** Principal component analysis of seven *Phragmites australis* descriptors.

	Eigen Vectors	
Component Number	PC1	PC2
Standard Deviation	2.09	1.11
Proportion of Variance (%)	61.84	17.49
Cumulative Proportion (%	61.84	79.33
	**Eigen Values**	
Plant_height	−0.33	-0.02
Stem_diameter	−0.45	-0.22
Fresh_flowers	−0.40	0.37
Dry_flowers	−0.41	0.31
Internode_distance	−0.30	0.48
Leaf_length	−0.41	-0.31
Leaf_width	−0.31	-0.62

**Table 4:** Microsatellite loci and allelic diversity measures in invasive and native *Phragmites australis* populations from Quebec, Canada and Kashmir, India.

	Loci	Allele Size Range (bp)			Number of Alleles			PIC
		KE	QE	QN1	KE	QE	QN1	
	PaGT 4	291–295	293–297	285	1–3	1–3	1	0.8
	PaGT 14	191–209	201–208	199	2–3	1–3	1	0.9
	PaGT 8	192–196	194–196	196	1–3	1–2	1	0.7
	PaGT 13	225–229	227–229	227–237	1–3	1–3	1	0.5
	PaGT 9	209–247	209–229	231	2–4	1–2	1–2	0.9
	PaGT 11	160–166	160–166	160–162	2–6	1–3	1	0.4
	PaGT 22	201–222	190–226	201–215	1–4	1–3	1–2	0.9
	PaGT 12	183–189	183–190	183–190	1–4	1–3	1–2	0.9
	PaGT 16	248–325	275–309	282	1–4	1–4	1	0.9
Mean								0.8

PIC: Polymorphic information content; KE, QE, QN are invasive haplotype from Kashmir, invasive haplotype from Quebec, and native haplotype from Quebec, respectively.

**Table 5:** Population genetic diversity parameters estimated in 19 invasive *Phragmites australis* populations of Quebec (Canada) and Kashmir (India) and one native population of the species in Quebec, based on nine microsatellite markers.

	Popn	*n*	*P*	*A*	*A_p_*	*A_R_*	*H*_O_	*H*_E_
	KE1	24	100	4.22	2.82	2.39	0.693	0.595
	KE2	24	77.78	2.00	2.04	1.82	0.689	0.434
	KE3	24	88.89	2.33	2.29	1.98	0.715	0.511
	KE4	24	100	3.33	2.63	2.16	0.722	0.543
	KE5	24	100	3.78	2.72	2.25	0.737	0.577
	KE6	24	100	4.56	3.37	2.54	0.779	0.665
	KE7	24	100	3.89	2.43	2.15	0.641	0.543
	KE8	24	100	3.78	3.10	2.4	0.731	0.598
	KE9	24	66.67	2.11	2.17	1.81	0.569	0.406
	QE1	24	88.89	3.56	2.16	2.06	0.375	0.445
	QE3	24	88.89	2.44	1.79	1.81	0.463	0.380
	QE4	24	100	2.56	1.93	1.95	0.495	0.441
	QE6	24	88.89	2.44	2.00	1.89	0.05	0.451
	QE7	24	100	3.00	1.92	1.94	0.463	0.408
	QE8	24	88.89	2.33	1.96	1.89	0.509	0.445
	QE9	24	88.89	2.44	1.80	1.77	0.393	0.354
	QE10	24	100	2.78	2.07	1.93	0.366	0.424
	QE11	24	100	3.56	2.32	2.18	0.579	0.521
	QE12	24	88.89	1.89	1.61	1.59	0.321	0.291
	QN1	24	88.89	2.22	1.72	1.54	0.228	0.204
Overall Mean		24	92.78	8.56	4.16	2.00	0.525	0.461

Abbreviations: Popn = population name; *n* = sample size; *P* = percentage of polymorphic loci; *A* = mean number of alleles per locus; *A_p_* = mean number of alleles per polymorphic locus; *A_R_* = Allelic richness; *H*_O_ = mean observed heterozygosity within populations; *H*_E_ = mean expected heterozygosity within populations diver.

**Table 6:** Geographic locations of *Phragmites australis* populations that were sampled in a study assessing and comparing the phenotypic and genetic diversity of the species in Quebec and Kashmir (India) with their putative population type as identified by morphometric characters (KE: alien invasive haplotype from Kashmir (India); QE: alien invasive haplotype from Quebec (Canada); QN1: native haplotype from Quebec; * populations that were sampled for phenotypic study; ‡: populations that were sampled for genotypic study; a.s.l = above sea level).

Region	Site of Collection	Population	Type	Latitude (N)	Longitude	Elevation (m a.s.l)
**Kashmir**	Forshore	KE1 *^‡^	invasive	34°5′19″	E 74°30′44″	1597
	Ashaibagh	KE2 *^‡^	invasive	34°4′11″	E 74°30′5″	1579
	Nageen	KE3 *^‡^	invasive	34°3′51″	E 74°29′59″	1595
	Rangharstop	KE4 *^‡^	invasive	34°3′46″	E 74°29′15″	1583
	KuhumusWullar	KE5 *^‡^	invasive	34°12′1″	E 74°21′35″	1580
	Saderkotewullar	KE6 ^‡^	invasive	34°13′40″	E 74°27′43″	1578
	Shalimar	KE7 ^‡^	invasive	34°50′5″	E 74°30′52″	1583
	Dal Lake	KE8 ^‡^	invasive	34°37′19″	E 74°30′57″	1585
	Ganderbal	KE9 ^‡^	invasive	34°8′9″	E 74°27′57″	1572
Quebec	La Pocatière	QE1 *^‡^	invasive	47°20′27″	W 70°5′41″	6.7
	St Grégoire	QE3 *^‡^	invasive	46°14′27″	W 72°33′6″	32.3
	Ange - Gardien	QE4 *^‡^	invasive	45°21′49″	W 72°55′50″	97
	Eastman	QE6 *^‡^	invasive	45°17′53″	W 72°18′32″	273
	Princeville	QE7 *^‡^	invasive	46°11′45″	W 71°54′40″	123.7
	Bernière	QE8 *^‡^	invasive	46°41′47″	W 71°18′13″	76.8
	Lac St Francois	QN1 ^‡^	Native	45°2′29″	W 74°27′47″	47
	Lac St Louis	QE9 ^‡^	invasive	45°23′57″	W 73°45′43″	28.6
	Parc Louis Racine	QE10 ^‡^	invasive	45°30′54″	W73°18′16″	20.3
	Sablière Colette	QE11 ^‡^	invasive	45°54′36″	W73°10′35″	8
	La Pocatière	QE12 ^‡^	invasive	47°20′27″	W70°5′46″	6.7

**Table 7:** Traits measured and methods that were used in a study aimed at investigating the phenotypic and genetic diversity of *P. australis* in Kashmir and Quebec.

Traits	Method Used
**Stem diameter**	Stem diameter was determined by measuring stem circumference at 25 cm above the ground surface using a metre tape graduated in 0.1 cm increments.
**Plant height**	Plant height was measured from soil surface to the apical node of the tallest shoot in each plant using measuring ruler tape.
**Leaf length and leaf width**	Leaf length and leaf width were measured at 2 cm from the axil of leaf using plastic measuring scale. These measurements were taken six times per individual plant
**Internode length**	Internode length was measured (±1 mm) from top to bottom of the plant using plastic measuring scale. These measurements were taken six times per individual plant
**Fresh mass of flowers and seeds**	Flowers and seeds from the entire seed heads were cut off from each individual plant were sampled to measure fresh mass flowers and seeds using a portable electronic scale that was graduated to 0.001 g (Mitutoyo, Japan)
**Dry mass of flowers and seeds**	The collected flowers and seeds were oven-dried at 70 °C for 3 to 4 days to determine the biomass of entire seed head from individual plant using a portable electronic scale that was graduated to 0.001 g

**Table 8:** Microsatellite loci optimized for population genetics analysis.

Conditions	Loci	^1^ Primer Sequences (5′–R′)	Dyes	PCR Protocol Tested	T*_a_* _(°C)_
Multiplex 1	PaGT 4	F: TGCTCCCTGCCAGTTTCTTG	VIC	McCormick et al. (2010) [61]	55
		R: TATCCACCCTTCGAAGGCAC			
	PaGT 14	F: GTTGCAGCAAGTATTTGG	VIC	Idem	55
		R: CAAGCATTCTAGTAGTAGC			
Multiplex 2	PaGT 8	F: TCTGAACATAATCCTGGTGG	FAM	Idem	55
		R: TCTGTGTGAAGCAGTTCTGC			
	PaGT 13	F: CTCATGCATCACTTCACAGG	FAM	Idem	56
		R: ACACGGACCTAACATCAACC			
Simplex 1	PaGT 9	F: CCATGTGTTAATGTTGTCC	PET	Idem	55
		R: ATTGAATCCACACGTTTCCG			
Simplex 2	PaGT 11	F CAACTCCGTGAATGACATGC	NED	Belzile et al. (2010) [60]	56
		R: CAGTTTGTGCACTAATGGAC			
Simplex 3	PaGT 22	F: TTGAGTGCCTGGTGTATTCG	VIC	Idem	56
		R AAGCTTCTGTCATGGAACCG			
Simplex 4	PaGT 12	F: CTTCCTAGGTCAGTATCATCC	PET	McCormick et al. (2010) [61]	55
		R: GTGGCAGCTGATTGATTTGG			
Simplex 5	PaGT 16	F: ACCAATCAGTCAGACTAGCC	FAM	Idem	56
		R: GTTCTCATGTTGGAGAAGCC			

^1^ Primer sequences were obtained from Saltonstall (2003a) [30].

## Data Accessibility

The primer sequences (PaGT8-PaGT22) that were used in the present study have GenBank Accession Nos. AY230868-AY230876. We agree to deposit our data sequences to a public repository.
